# Gut Microbial Changes, Interactions, and Their Implications on Human Lifecycle: An Ageing Perspective

**DOI:** 10.1155/2018/4178607

**Published:** 2018-02-26

**Authors:** Ravichandra Vemuri, Rohit Gundamaraju, Madhur D. Shastri, Shakti Dhar Shukla, Krishnakumar Kalpurath, Madeleine Ball, Stephen Tristram, Esaki M. Shankar, Kiran Ahuja, Rajaraman Eri

**Affiliations:** ^1^School of Health Sciences, College of Health and Medicine, University of Tasmania, Launceston, TAS 7250, Australia; ^2^School of Biomedical Sciences and Pharmacy, The University of Newcastle, Callaghan, NSW, Australia; ^3^Rehabilitation and Aged Care Services, Kingston Center, Monash Health, Monash University, Melbourne, VIC, Australia; ^4^School of Health and Biomedical Sciences, RMIT University, Bundoora, VIC 3082, Australia; ^5^School of Basic and Applied Sciences, Central University of Tamil Nadu, Thiruvarur, India

## Abstract

Gut microbiota is established during birth and evolves with age, mostly maintaining the commensal relationship with the host. A growing body of clinical evidence suggests an intricate relationship between the gut microbiota and the immune system. With ageing, the gut microbiota develops significant imbalances in the major phyla such as the anaerobic Firmicutes and Bacteroidetes as well as a diverse range of facultative organisms, resulting in impaired immune responses. Antimicrobial therapy is commonly used for the treatment of infections; however, this may also result in the loss of normal gut flora. Advanced age, antibiotic use, underlying diseases, infections, hormonal differences, circadian rhythm, and malnutrition, either alone or in combination, contribute to the problem. This nonbeneficial gastrointestinal modulation may be reversed by judicious and controlled use of antibiotics and the appropriate use of prebiotics and probiotics. In certain persistent, recurrent settings, the option of faecal microbiota transplantation can be explored. The aim of the current review is to focus on the establishment and alteration of gut microbiota, with ageing. The review also discusses the potential role of gut microbiota in regulating the immune system, together with its function in healthy and diseased state.

## 1. Introduction

The human body contains a diverse range of bacterial species, with lesser representation from viral and eukaryotic microbes, that have been referred to as a “microbial bank.” As the human gut harbors most of the microbes, it can be considered as a “microbial organ.” All the microbes in the gut are collectively known as “gut microbiota” and genomes associate with them represent the “gut microbiome.” Previously, the bacterial count in a healthy adult was estimated to be 10^14^ to 10^15^ cells. However, recently revised [1], the bacterial load in a healthy adult (using volume and mass as variables) was estimated to be approximately 3 × 10^13^ bacterial cells in a 70 kg adult. The microbiota is not a homogenous population of microorganisms; rather, it is comprised of an intricate range of microbial communities that interact with each other as well as with the host, in a way that impacts the health of the host [2]. The Bacteroidetes and Firmicutes are the major bacterial phyla, with subgroups like Fusobacteria, Cyanobacteria, Proteobacteria, Verrucomicrobia, Actinobacteria, and a few others.

A healthy intestinal tract is relatively stable throughout the adulthood, but with the ageing process, perturbations occur with exogenous factors such as antibiotics use and diet and endogenous factors like cellular stress. Ageing and related complications are leading public health concerns worldwide [3]. Ageing is accompanied by major physiological changes such as alteration in the gut microbial composition (dysbiosis), immune responses, and metabolism which may lead to various gastrointestinal (GI) tract related inflammatory conditions, and autoimmune disorders [4, 5]. The usual threshold age for defining older adults or elderly is above 63–76 years, and around this age the gut microbiota loses its relative stability [6, 7]. Any compositional differences with ageing have a direct effect on the intestinal motility and digestion [8]. Fermentation processes in the colonic gut are altered adversely with variations in the microbiota. This affects the homeostasis in the gut, leading to immunosenescence (decline in immune responses) and inflamm-ageing, that is, low-grade inflammatory response [9–11]. In the present review, we focus on the establishment and alteration of gut microbiota with ageing. Moreover, the review also discusses the potential role of microbiota in regulating the immune system, and its function in healthy and diseased state.

## 2. Microbiota and Ageing

### 2.1. Infancy

The sterile period (in the human life cycle) is during the gestation, where the fetus grows in the uterus and remains tolerant to maternal antigens. However, a few studies have confirmed the presence of some bacteria in the amniotic fluid in the uterus, but the number and diversity were found too low to show any effect on infant gut colonization [2, 12].

**Figure 1:**
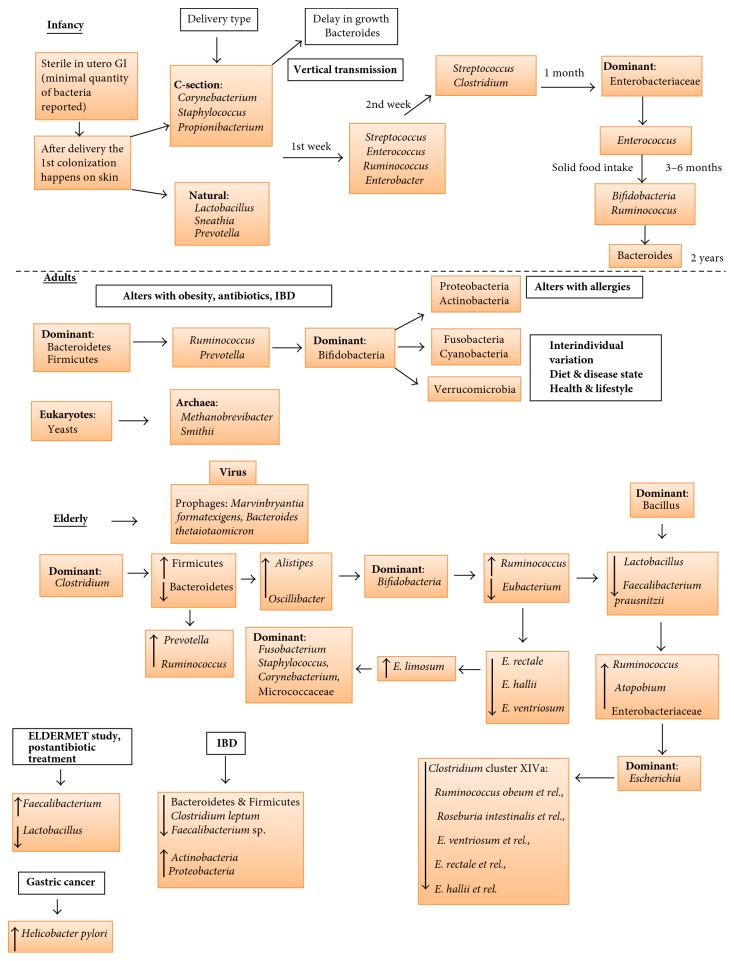
*Overview of development of microbiota*. The gastrointestinal tract (GI) is most sterile during the in utero stage. The first colonization happens based on mode of delivery either C-section or natural (vaginal delivery).* Corynebacterium *sp. is thought to be early colonizers in C-section and* Lactobacillus* sp. in the vaginal delivery. As the time progress the commensal bacterial community grows and is influenced by the solid food intake. During the initial stages of microbiota establishment the TLR receptor actions are minimal allowing growth of commensals. Eventually the immune system also grows by demarking the commensals and pathogens. Bacteroidetes domination begins after two years of birth. The relative stability is attained at the adulthood with Bacteroidetes and Firmicutes dominating. The alteration happens with use of antibiotics, obesity, GI orders, and diet. During elderly the relative stability declines, commensal community reduces, and pathogenic species like* Clostridium* increases. Malnutrition, alcohol abuse, decline in metabolism, frequent hospitalization, nosocomial infections* (Clostridium difficile),* and other pathogenic infections leading to Polypharmacy and ultimately to various inflammatory diseases.

The initial colonization on the skin from environmental organisms takes place immediately after delivery, followed by gut colonization influenced by maternal and dietary factors. The nature and extent of neonatal colonization are also influenced by the method of delivery, whether vaginal or via the use of instrumental procedures (Figure 1). During vaginal delivery, studies demonstrated the presence of* Lactobacillus, *followed by* Prevotella* and* Sneathia* species [2, 23, 24]. After a caesarean section (C-section),* Staphylococcus*,* Propionibacterium*, and* Corynebacterium* species and high numbers of* Clostridium difficile *and* Escherichia coli* were found in abundance in the gut compared to infants delivered vaginally [25, 26].

The possible basis of the bacterial communities establishment in the gut has been described in a study [24] where they followed two healthy infants from birth for 10 months. Screening using faecal samples and polymerase chain reaction and denaturing gradient gel electrophoresis (PCR-DGGE) identified* Streptococcus thermophiles, Ruminococcus gnavus, *and* Enterococcus raffinosus *predominantly throughout the study. In the 3rd and 4th day faecal samples, there was rapid colonization with bifidobacterial species which remained strong for 3 months in both the infants. Beyond 3 months,* Clostridium *species colonized dominantly, which contradicted the culture-based theory. According to the culture-based evidence,* Enterobacterium* (gram positive) was the first colonizer, which created and maintained the stable environment for Bacteroidetes,* Bifidobacterium,* and* Clostridium *to grow in infants. However, the research, either culture-dependent or independent, explains that the gut colonization in infants exhibits low diversity, instability, and high dynamics [27, 28]. These experiments were conducted using DNA from faecal samples of 13 infants 1 month, 3 months, and 7 months old, which indicated the gut microbiota is stable over this time frame, though there was interindividual variability [29]. Antibiotics administration has an influence on infant gut colonization. Infants who were administered with antibiotics have lower proportions of lactic acid bacteria and enterococci [1].

Besides antibiotics use, diet is also known to play a major role in the gut microbial colonization. The gut of infants who were breast-fed predominantly comprised streptococci, bifidobacteria, and* E. coli*. Enterobacteria and* Bacteroides* were common in the gut of infants who were formula-fed [30]. Changes occur to gut colonization after introduction of solid foods. A study compared the influence of diet on the gut microbiota in children (1 to 6 years) who consumed the Western diet and compared to African diet (fibre-rich intake). After the Western diet, children had reduced microbial diversity with lower levels of Actinobacteria and Bacteroidetes compared to African diet.

### 2.2. Immunity and Initial Colonization

The mucosal immune system and gut microbiota coevolve with age. A few studies indicate that the development of innate and adaptive immune systems require microbial interactions during infancy [31, 32]. A study also suggested the role of delivery mode in the development of immunological functions and gut microbiota [33]. This study revealed that infants born by C-section have relatively higher levels of immunoglobulins produced by peripheral blood components compared to infants born by vaginal delivery. Immunoglobulin A (IgA) is mainly secreted by the gut mucosal layer which contributes to gut barrier function. IgA elicits low-grade immune responses allowing bacteria to colonize in the gut [34]. The corelation between immunoglobulins and gut microbiota development in gnotobiotic mice was investigated by Planer et al. [35]. Briefly, to determine age-related differences in IgA response, donor microbiota (infants) was introduced into mice, and mice faecal samples were collected. Interestingly, the IgA responses were similar to those of the infant donor population. This study can be used as an indicator for understanding the gut mucosal immunity and microbiota in health and disease. IgA plays a major role in mucosal immunity, as it is induced in response to colonization by specific commensal bacteria to protect the mucosal surfaces and contributes to the host-microbiota mutualism.

### 2.3. Childhood, Preadolescence, and Adulthood

In early childhood (between 1 and 5 years), the expansion of bacterial diversity slows down, and the gut microbial diversity remains lower compared to adults. The gut microbiota in childhood is more stable and dominated by multiple members of Bacteroidetes. In the healthy preadolescence (7 to 12 years), the gut microbiome is species rich, containing many bacterial taxa and functional genes similar to adult microbiota enriched with Lachnospiraceae,* Anaerovorax*,* Bifidobacterium*, and* Faecalibacterium*. The bacterial composition in adults is predominantly comprised of Bacteroidetes and Firmicutes (Figure 1). It has been shown that the relative abundance of both Bacteroidetes and Firmicutes can vary between 0 and 99% [2, 36]. Other groups of researchers reported the presence of bacteria from phyla Proteobacteria, Actinobacteria, Fusobacteria Cyanobacteria, and Verrucomicrobia, as well as methanogenic archaea, multiple phages and Eucarya in healthy individuals [14, 37–39]. At phyla level, the gut microbiota in adults is stable as compared to infants; however, there is a significant variation in specific microbial species and their proportions. There is no detailed study demonstrating microbial composition of healthy adults due to vast interindividual variation in composition. This variation can be linked to environmental factors shaping microbiota immediately after birth and low temporal variation and also due to genetic variation. A holistic approach to identify conserved and similarities among healthy adults by proposing the presence of enterotypes such as* Bacteroides* (enterotype I),* Prevotella* (enterotype II), and* Ruminococcus* (enterotype III) [26]. They have been shown to remain relatively stable for around 6 months and evidence of fluctuations between enterotypes was revealed in a 10-year follow-up study [40]. Despite taxonomic variability, the functional properties of the adult gut microbiota are relatively consistent with pathways involving metabolism and fermentation. In older adults, the gut microbiota becomes less diverse and unstable due to coexisting conditions and age-related factors.

### 2.4. Older Adults

Imahori (1992) described ageing as “regression in physiological functions followed by advancement in age” [41]. Conventionally, people of age 65 years and above are termed as older adults by chronological measurement [7, 42, 43]. Understanding gut microbiota and its modulations is an essential factor in improving the health and the well-being of older individuals. Perturbations in the gut microbial composition are associated with chronic conditions such as obesity and inflammatory diseases. The interindividual variability is much higher in the older adults than in adults or infants. There is a significant dysbiosis in gut microbiota with age, together with the use of antibiotics and lack of nutrition. A reduction in mastication ability with less salivary function and loss of dentition can limit the nutrient intake and, thus, impact the microbial growth. Oropharyngeal and esophageal motility are reduced with ageing resulting in swallowing propulsions and lowered esophageal sphincter pressure, leading to an increased prevalence of gastroesophageal reflux. With age, there is also an increased intestinal transit time due to reduced motility, which can reduce digestion and absorption, and with reduced appetite, this may lead to malnutrition [7, 44]. In particular, hypochlorhydria is associated with ageing and is prevalent in those who have or previously had* Helicobacter pylori* infection. Hypochlorhydria predisposes to malabsorption, alteration in bacterial growth, and vitamin B12 deficiency leading to atrophic gastritis (autoimmune), and loss of parietal cells. Malnutrition is one of the key factors affecting the growth of gut microbiota and contributes to an impaired immune system in the older adults.

### 2.5. Specific Gut Microbial Changes in the Older Adults

A significant decrease in the relative proportions of Bacteroidetes and Firmicutes in the older adults, when compared to adults, was observed in a study by Mariat et al. [18] (Figure 1). Apart from these major two phyla, a decline in* Clostridium* cluster IV was also observed in institutionalized older individuals [45]. The relationship between frailty scores and diversity of the microbiota in the older people was shown in a study that involved 23 older volunteers and analysis of their faecal samples [46]. A substantial decrease in Bacteroidetes,* Prevotella, Lactobacillus, Candida albicans, Streptococcus, Staphylococcus, *and* Faecalibacterium prausnitzii* and an increase in levels of* Ruminococcus, Enterobacterium,* and* Atopobium* was reported [18, 46]. The relative variability of gut microbiome among the older population was measured and analyzed by Claesson et al. [45]. Faecal samples from 165 older people and 9 young (controls) volunteers were collected and microbiota analysis was performed using 16S ribosomal RNA (rRNA) gene sequencing technology. The proportions of Bacteroidetes and Firmicutes in individuals varied from 3% to 92% and 7% to 94%, respectively. A study on the faecal bacteria in healthy old volunteers (age range 63 to 90 years; *n* = 35) living in the local community, old, hospitalized patients (age range 66 to 103 years; *n* = 38), and old, hospitalized patients receiving antibiotic treatment (age range 65 to 100 years; *n* = 21) [47] exhibited a decrease in* Bacteroides-Prevotella* group in the old, hospitalized patients. Another study investigated the age-related changes in the gut microbiota and host immune system among young adults (average 30 years), older adults (65 to 75 years), and centenarians (99 to 103 years) using the Human Intestinal Tract Chip and 16S rRNA gene sequencing methods [6]. Young adults and the older adult group showed a very comparable gut microbiota with Bacteroidetes and Firmicutes, highly dominant, and small proportions of Proteobacteria and Actinobacteria. In contrast, there were significantly higher levels of Proteobacteria in the centenarian population, relative changes in Firmicutes subgroups with a decrease in* Clostridium* cluster IV, and an increase in* Bacillus *species. The microbiota differences between adults and the older adults in four different European countries (Italy, France, Sweden, and Germany) were studied by [16] and found to be country specific. Other studies also found differences in the gut microbiota with respect to age, gender, and geographical locations [16, 48].

Recent findings suggest that the gut microbial composition differs in men and women [36, 49]. Another study compared gut microbiota of obese and lean men and women (mean age of 60 years) with the same nutritional intake [50]. This study revealed higher levels of Firmicutes in women regardless of age and body mass index (BMI), and men had lower levels of* Bacteroidetes* than women with BMI over 33 kg/m^2^. The differences in the gut microbiota between men and women may be influenced by the grade of obesity and this could help in understanding the different prevalence of metabolic and GI diseases between males and females. The ELDERMET study by Claesson et al. [45] compared the microbial composition of older and young adults and found characteristic differences in Bacteroides and* Clostridium* levels. There was greater variability in microbial composition in the older people potentially contributing to greater morbidities (Table 1).

### 2.6. Metagenomic and Metabolic Changes Associated with Gut Microbiota in Ageing

Mounting evidence indicates that gut microbiota influences metabolism [51–53] and may play a significant role in triggering metabolic diseases [54]. The relationship between microbiota and metabolic and functional pathways in the humans was investigated by The Human Microbiome Project (HMP). From the HMP, it is known that the human microbiome is highly variable both within a single subject and between different individuals. During the ageing process, the host is challenged by various changes, including diet and concomitant exposure to multiple medications, including antibiotics, reduced physical activity, and/or any underlying disease, which forces microbiome reshuffling to adapt to the change [55].

A study investigated the functional differences between the gut microbiome across age groups, including young adults, older adults, and centenarians [6]. The study characterized the metabolic trajectory of the gut microbiota metagenome using illumina shotgun sequencing on 9 faecal samples. It was found that proteolytic activity was increased and there was a clear loss in the genes associated with the metabolism of carbohydrates upon ageing. The capacity of short chain fatty acids (SCFAs) production also declined due to the age-related reduction of genetic pathways caused by overall rearrangement of the microbiome. The data on differential abundance in microbiome indicated structural and functional changes in microbiota in the aged population, moving from saccharolytic to a putrefactive metabolism. The study also found that many shifts in the microbiome were forcing rearrangements in the core metabolic potential of the centenarians intestinal microbial ecosystem. This specific microbiome analysis allows us to assess the role of microbiota in pathophysiological conditions in the older people cohort.

Manipulating the intestinal microbiota and microbiome may be beneficial for maintaining health and treating certain disorders, particularly common among the older individuals. Nevertheless, more comprehensive clinical studies in different age groups are required to fully understand the role of microbiome in metabolism.

### 2.7. Circadian Rhythm, Microbiome, and Metabolism in Ageing

Circadian rhythm, metabolism, and gut microbiota are intricately linked. A study compared human sleep-wake cycle and insulin secretions and found robust variations in glucose regulation during normal and no sleep conditions [56]. Most of the glucose tolerance occurs during sleep which influences the nocturnal brain and tissue glucose utilization. Therefore, chronic sleep disturbances and sleep apnea in the older individuals may be linked to alterations in metabolism. With reduced physiological function and sleep patterns, the older adults may exhibit altered appetite leading to increased susceptibility to GI and other metabolic disorders. Bass and Turek [57] demonstrated the association between sleep-wake cycle and metabolic regulation and suggested that interventions in sleep disorders may ameliorate some of the metabolic deficits associated with overweight and obesity [57, 58]. Another study critically reviewed circadian rhythm, sleep, and metabolism [59]. They suggested an association between clock gene variations, obesity, and metabolic functions in understanding the impact of circadian rhythm. Similarly, a study demonstrated a bidirectional relationship between circadian clock and metabolism, tested in a high fat diet animal model [60]. A research scholar hypothesized that the gut microbiota and microbiome are one of the key elements in maintaining the circadian rhythm impacting the dietary and physiological functions of the body [61]. To test their hypothesis, they used germ-free mice and specific-pathogen free mice. The study highlights the relation between diet, gut microbial function, metabolome, and metabolic function impacting the host health. The authors speculated that any change in diet impacts the circadian rhythm and manipulation in the gut microbial structure might restore the metabolic balance. Age-related perturbations in gut microbial structure and microbiome caused by diet and other factors appear to affect the circadian clock, promoting metabolic disorders and obesity. The relationship between the gut microbiota and metabolism was first shown in germ-free mice compared to conventional mice (both groups on a high fat diet) [62]. Conventional mice gained weight (as expected), whereas the germ-free mice maintained their body weight, suggesting an impaired feeding efficiency in the germ-free mice. Studies by Turnbaugh et al. [62] confirm this hypothesis, when germ-free mice gained weight when colonized with the microbiota from obese rather than from lean mice. The possible mechanism could be the activation of the non-insulin dependent AMP-activated kinase pathway, which controls energy expenditure via the increase in glucose oxidation under metabolic stress conditions. Interestingly, intestinal dysbiosis was observed in the obese leptin-deprived* ob/ob* mice and it was subsequently shown that there was microbial dysbiosis in obese humans when compared to lean controls, suggesting a common mechanism in mice and humans [51]. Therefore, gut microbiota can be considered dependent on the host genome like observed in the* ob/ob* mice. Importantly, the role of the gut microbiota in maintaining homeostasis could also play a part in the microbial genome [63].

## 3. Role of Gut Microbiota in Immune Function in the Older Adults

### 3.1. Pro- and Anti-Inflammatory Responses

**Figure 2:**
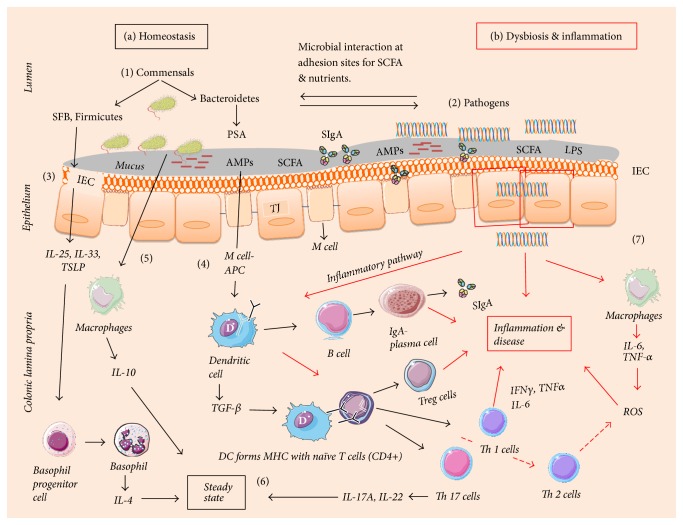
*Interplay between immune system and gut microbiota in homeostasis, tolerance, and inflammation*. (a) Both commensals and opportunists compete for the metabolites (SCFA) and various nutrients. The intestinal epithelial cells (IEC) play a role in steady state environment by releasing interleukins IL-25, IL-33, and thymic stromal lymphopoietin (TSLP) factors in the presence of SCFA, PSA* (Bact., fragilis)*, lipopolysaccharide (LPS), and AMPs (defensins, cathelicidins, and C-type lectins). IL-25, IL-33, and various growth related factors help in the transformation of progenitor basophils to basophils, activation of monocytes, macrophages, and mast cells functioning. The bacteriocins released by segmented filamentous bacteria (SFB) also influence the release of TSLP. The microfolding (M) cells upon sensing the presence of the microbes act as antigen presenting cell (APC) phagocytic activity by engulfing and presenting it to mucosal dendritic cells (DCs). In turn DCs are endowed with the ability to produce cytokines and other products such as IL-6 and IL-1*β*, tumor growth factor (TGF-*β*), retinoic acid (RA), and vitamin A. DCs form a major histocompatible complex (MHC) with the T cell receptors (TCR). In the presence of TGF-*β* and RA, the naïve T cells (CD4+ cells) transform into regulatory T cells (Treg). Simultaneously during interaction and competition of commensals and pathogens for nutrients, macrophages after recognition microbes released proinflammatory cytokine such as IL-10, which in turn helps with expansion of Treg cells which are already released during homeostasis and inflammation. Also with the help of DCs, macrophages release certain B cell activating factors which increase the production of secretory immunoglobulin A (SIgA) to maintain tolerance and steady state. (b) ((1) & (2)): in the elderly, there are declined physiological functioning and dysbiosis (reduction in commensal bacteria), resulting in an increase in pathogens. (3) The production of IL-25, IL-33, and thymic stromal lymphopoietin (TSLP) by IEC reduces. (4) There is decline in M cell/APC activity to present PSA or microbe to DCs (activation of inflammatory pathway). Moreover lack of PSA stimulation reduces the IL-12 levels and releases T helper 2 cells. Decline in DCs not forming MHC with TCR reduces the population of active T cells such as Treg cells. Activation of B cells to plasma secretory cells and release of SIgA decreases. (5) The activation and function of macrophages (low levels of IL-10) are reduced. (6) The steady state or the tolerance is reduced. (7) Macrophages (inflammatory) are activated in the presence of pathogens and release proinflammatory cytokines (IL-1*β*, IL-6, and TNF-*α*) which leads to production of reactive oxygen species (ROS) and causes oxidative stress. Altogether with reduced levels of Treg and T helper cells and SIgA increase the pathogen invasion leading to release of proinflammatory cytokines and reduced anti-inflammatory cytokines increases inflammation, causing various GI disorders.

The gut associated immune system is the largest component (approximately 70%) of the human immune system [64]. Along with numerous commensal bacteria, there are abundant innate and adaptive immune cells in the gut (Figure 2). Gut microbiota and the immune system actively interact to maintain the homeostatic equilibrium [65]. This equilibrium depends on the intestinal epithelial cells (IEC) in the colon to segregate microbes and mucosal immune cells. Apart from IECs, enterocytes and gut associated lymphoid tissue (GALT) form a specialized barrier against invading pathogens. The goblet cells of the IECs in the colon are capable of secreting gel-like layers of mucus such as mucin and mucin 2 (MUC 2) which forms the first line of defense against the microbial breach [66, 67]. Trefoil factor 3 (TFF3) and Resistin-like molecule-*β* (RELM*β*) are other goblet cell derived products that contribute to forming a physical barrier in the intestine. During inflammation, RELM*β* helps to secrete MUC 2 and regulate macrophages and adaptive T cells [68]. Antimicrobial peptides (AMPs) secreted by enterocytes present in the IEC further bolster the protective barrier. For example, in the presence of* B. thetaiotaomicron* and* L. innocua* or stimulation with lipopolysaccharide (LPS), the AMP levels were high in the IECs [69]. Enterocytes also possess specialized pathogen pattern recognition receptors (PPR) which contain Toll-like-receptors (TLRs) and nucleotide oligomerization domain-like-receptors (NOD), which recognize bacterial surface molecular structures called microbe-associated molecular patterns (MAMPs). MAMP recognition leads to activation of an inflammatory response causing the release of nuclear factor kappa B (NF-kB) and tissue damage and NOD recognizes the damage-associated molecular patterns (DAMPs) in the host [70]. All the TLRs are present at the mRNA level of IEC with different concentration levels. TLRs 2, 4, 5, and 9 detect the presence of bacterial and fungal pathogens and TLRs 3, 7, and 8 detect viral pathogens in the small and large intestine. Microfolding (M) cells present in the subepithelial region with lymphocytes and dendritic cells (DCs) at the base open into the lamina propria (LP). M cells engulf pathogens by phagocytosis or endocytosis and present the antigen (APCs) to DCs. DCs, in the presence of a high number of interleukins (IL-1*β*, IL-6) and transforming growth factor (TGF-*β*), which unify the immune responses towards T helper (Th) 17 cells, then trigger widespread inflammation by the release of IL-17A, IL-17 F, IL-21, IL-22, and IL-23 [71]. Thus, DCs trigger both pro- and anti-inflammatory responses in the host.

### 3.2. Role of Enterocytes

Microorganisms are generally located on the outer mucosal layer of the epithelium in a compartmentalized fashion in the lumen. In order to reduce the interaction of microbiota and enterocytes, there is a dense gel-like inner layer on top of the epithelium secreted by goblet cells (MUC 2) which is devoid of microbes where in contrast the outer layer is thin and colonized with bacteria [72]. Enterocytes sense the presence of microbes within the mucus layers and monitor their proximity and density. Thus, enterocytes act as the frontline in the microbiota-immune cells crosstalk that occurs in the host. In healthy ageing, enterocytes monitor GALT immune response, send tolerance signals towards commensals, and keep DCs in a stable mode [73]. M cells (APCs) present the antigen to naïve CD4+ cells, causing their differentiation into CD4+ T cells (T-regulatory), which elicit an anti-inflammatory response (IL-10, TNF-*α*). Concurrently, the antigens are also presented to B cells in LP, stimulating the differentiation of immunoglobulin A (IgA). IgA helps in neutralizing the toxins produced by microbes and prevents the adherence of the microbiota and the intestinal lumen. Secretory IgA (SIgA) induction appears more efficient in the presence of Bacteroidetes [74].

In the older adults, the preclinical and clinical studies indicate a decline in immune function [65, 75, 76]. In aged IEC (GALT and enterocytes), impairment and changes in immune functions have been observed in animal models [72]. With GALT impairment, there is a reduction in mucin and defensins (*α*-defensins and*β*-defensins) leading to an increased chance of infection by uncontrolled growth of commensals (mutualistic to opportunistic) and pathogens. When there is an increase in pathogens, enterocytes could possibly activate certain cytokines and chemokines forcing DCs to initiate proinflammatory response by differentiation of Th-1, Th-2, and Th-17 (effector) cells [77]. A study by [78] demonstrated that* Bacteroides fragilis* releases polysaccharide A (PSA) recognized by TLR and induces production of IL-10 by Treg cells (in the presence of TGF-*β*). Induction of Treg cells is not restricted to* Bacteroides fragilis*, as the presence of an indigenous* Clostridium* species also promotes Treg cell activation. A study [79] showed the role of clostridia in inducing Treg cells in germ-free mice. A total of 17 strains from Clostridiales clusters VI, XIVa, and XVIII isolated from human faeces exhibited Treg-inducing activity, suggesting that* Clostridium*-dependent induction of Tregs may contribute to the maintenance of intestinal immune homeostasis [21]. A change to the Bacteroides-Firmicutes group triggers the release of IL-6 by DCs in the LP and activates naïve T cells. Naïve T (T0) cells differentiate into Th-17 and Th-1 cells, which are responsible for inflammation in colitis [80].

### 3.3. Short Chain Fatty Acids (SCFAs)

Gut microbiota generate a range of metabolites, particularly SCFAs such as butyrate, propionate, and acetate. These SCFAs are linked to the elicitation of anti-inflammatory and antineoplastic responses via inhibition of NF-kB favoring growth of the intestinal epithelium [81]. Butyrate is considered the primary source of energy to the intestinal epithelium and helps in the production of mucin (gel-like inner layer) in the lumen which establishes the physical barrier. Deficiency of SFCA leads to epithelial atrophy and inflammation during colitis [82]. In the older adults, the decline in butyrate levels is correlated to a decrease in* F. prausnitzii*,* Eubacterium hallii,* and* Eubacterium rectal* [6]. Also, due to the decline in butyrate and weakened physical barrier, the immunologic tolerance decreases, allowing the increase in Enterobacteriaceae in the older adults. This was corroborated in a study conducted by [6], where proinflammatory cytokine levels increased and were related to inflamm-ageing. Additionally, reduction in proinflammatory responses and decline in SCFA levels have been linked to risk of colorectal cancers [83, 84].

### 3.4. Sex Related Differences

Both the immune and the endocrine system exhibit significant sex-specific differences. A transcriptome study on peripheral blood mononuclear cells revealed lower T cell responses and more inflammation in older females [85]. Another study from Japan on an ageing population indicated progression of immunosenescence in older men with a decline in T cell proliferation when compared to older women [86]. Age-related changes in sex steroid levels enhance immunosenescence-related alterations. It is also shown that gut microbial and sex hormone differences with ageing may lead to autoimmune diseases in animal models [87]. The association of sex differences, ageing, and immune response with gut microbiota was critically reviewed by [86]. The authors emphasized that early exposure to environment influences microbiome and sex-dependent immune responses and suggested that sex should be considered as a biological variable in immunological studies.

### 3.5. Dietary Influences

Due to reduced immune activity, the older adult population are generally categorized as immunocompromised as their resistance towards infections is lowered. The link between microbiota and the immune system can be demonstrated by examining the effects of the administration of various dietary supplements such as prebiotics, probiotics, and synbiotics (Table 2). Dietary supplementation of a probiotic drink with* Bacillus subtilis* at a rate of 2 × 10^9^ colony forming units (CFU) for 18 days (*n* = 44) increased the levels of SIgA by 65% in the stools and saliva during common cold infections in the older adults [88]. Consumption of a probiotic drink containing* L. casei *Shirota (LcS) at 1.3 × 10^10^ CFU for 4 weeks increased natural killer (NK) cell activity and significantly decreased intensity of CD25 in T resting cells in healthy non-immunocompromised older people [89, 90]. Prebiotics such as a bifidogenic growth stimulator and galactooligosaccharide when consumed with fermented milk for 4 weeks improved and maintained antibody titers for longer periods through improving intestinal microbiota in the older adults with influenza vaccination [91]. Similarly, in a randomized, double-blind study conducted on 43 older people, supplementation of a synbiotic (containing probiotic* Bifidobacterium longum* and an inulin-based prebiotic synergy 1) for 4-weeks increased the bifidobacterial count and also increased the counts of Actinobacteria and Firmicutes. Proteobacteria were reduced by 1.0 log units. There was reduction of proinflammatory cytokine TNF-*α* and an increase in butyrate production [75]. Therefore, dietary supplementation with pre/pro/synbiotics in the older adults not only increases the intestinal commensal diversity but also reinforces the immune system against various pathological conditions.

## 4. Modulation of Gut Microbiota for Health

As discussed in the earlier sections, diet plays an important role during the establishment and development of stable gut microbiota and can assist in its modulation. Due to the high prevalence of malnutrition in the older adults, dietary manipulation may play an important role. There is a decline in SCFA production in the older adult's gut due to a major shift in bacterial composition and malnutrition. This in turn contributes to a decline in anti-inflammatory response leading to an increase in infections. A high-fibre diet is widely recommended for adults and the older adults as it increases the SCFA production and decreases the intestinal pH, reducing the colonization of pathogenic bacteria. However, high fat diets with certain cooking oils containing polyunsaturated fatty acids (omega-3-PUFA) increased the levels of Firmicutes and Actinobacteria and decrease Bacteroidetes [91].

Many specific therapies have been proposed for treating dysbiosis, such as the use of prebiotics, probiotics, and synbiotics. A number of clinical studies demonstrated the ability of probiotics (most used are* Lactobacillus, Bifidobacterium, Lactococcus, *and* Streptococcus*) in modulating the gut bacteria in infants, adults, and the older adults [21, 92, 93]. These studies indicated an increase of SCFA production and improvement of the immune system [30]. Prebiotics alone were shown to improve the gut microbiota and help in production of SCFA [94].

Broad spectrum antibiotic therapy successfully treats many infections, however, at the expense of commensal bacterial loss. In all ages, careful use of antibiotics is preferred and when used, the potential use of narrow spectrum or targeted therapy is preferred, as this reduces microbiota alteration.

The concept of faecal microbiota transplantation (FMT) was introduced to control recurrent infections. FMT can be defined as a nature-tailored probiotic to control recurrent infections. Basically, a faecal sample from a healthy donor is selected and infused into the diseased patients via colonoscopy, endoscopy, sigmoidoscopy, or enema [95]. FMT produced 90% benefit as a treatment option for diarrhea due to recurrent* C. difficile* infection. In 2013 the United States Food and Drug Administration categorized faecal samples as a biological, investigational tool for the purpose of a therapeutic agent [93]. Many researchers have shown the effectiveness of FMT in IBD, Crohn's diseases, and ulcerative colitis, but important aspects of this treatment such as the screening and selection of healthy donors are still to be defined. FMT could also possibly be used against* H. pylori* infection (which may cause ulcers and gastric cancers), to minimize the effects of antibiotic use, particularly in the older adult population [96].

## 5. Conclusion

Gut microbial diversity declines with age and its function in metabolism and regulation of the immune system is reduced. This provides a chance for opportunistic pathogens to invade and inflame the gut giving rise to various diseases ranging from low-grade chronic ill health to those causing hospitalization and even death. Despite significant research on gut microbiota, the optimal therapy to reduce/prevent the dysbiosis in the older adults is yet to be identified. Diet plays a role as a manipulator of the gut microbiota throughout life and this may be particularly important in the older adults. The use of broad spectrum antibiotics almost certainly has an adverse effect on gut bacteria. We believe probiotic supplementation has significant potential to restore the diversity of the gut microbiota and improve immune function. However the specifics of supplementation, dosage and other parameters are still unclear. More well-conducted randomized studies on probiotics/prebiotics/synbiotics in the older adults are needed.

## Figures and Tables

**Table 1:** Changes in the gut microbiota in the healthy ageing (natural, non-antibiotic treated).

Study details	Sample details	Methods	Changes	Ref
Comparative study	*n* = 30 (i) Rural (high fibre diet) *n* = 15 median age 84 (ii) Urban (low fibre diet) *n* = 15 median age 68	Culture based analysis	(i) Rural: High in Bifidobacteria, Bacilli and mainly *Clostridium perfringens* (ii) Urban: Low *Bifidobacterium adolescentis *and *Fusobacterium mortiferum *strains	[13]

Analysis of gut microbiota	*n* = 6	16S rRNA gene sequencing, culture-based	(i) Bacteroides, Gamma-Proteobacteria and *Clostridium* IV, IX, XIVa dominated	[14]

Characterization of jejunal, ileal, caecal and rectosigmoidal colonic microbiota	*n* = 3 (i) Colon autopsy samples	16S rRNA gene sequencing	(i) Jejunal and Ileal microbiota: streptococci, lactobacilli, Gammaproteobacteria, the *Enterococcus* group and the Bacteroides (ii) Caecum: Bacteroides, *Clostridium* (iii) Sigmoid-colon: *C. coccoides* group, *the C. leptum* subgroup, the Bacteroidetes group, Gammaproteobacteria, the *Bifidobacterium*, streptococci and lactobacilli groups	[15]

Cross-sectional study	*n* = 230 (i) Sweden, adults (SA) *n* = 20, elderly (SE) *n* = 40 (ii) Germany (GA), adults *n* = 23, elderly (GA) *n* = 38 (iii) France, adults (FA) *n* = 22, elderly (FE) *n* = 27 (iv) Italy, adults (IA) *n* = 30, elderly (IE) *n* = 40	FISH and flow cytometry	(i) SA: *Eubacterium rectale-Clostridium coccoides *low in number High *Lactobacillus-enterococcus* group in both SA and SE (ii) GE: *Eu., rectale-C. coccoides *cluster higher (iii) FA: low levels of *Bacteroides-Prevotella* group. Low levels of bifidobacteria trend in FE (iv) IA & IE: lowest levels of *Lactobacillus-enterococcus* group. High *Bifidobacterium *group (v) High enterobacteria levels in all countries in the elderly	[16]

Observational study	*n* = 249 (i) Infants *n* = 150, age 1 to 12 months; (ii) Adults *n* = 54, age 25 to 35 years; (iii) Elderly *n* = 45, age 80 to 82 years.	16S rRNA gene sequencing and FISH analysis	(i) *Akkermansia muciniphila* is present and colonizes the intestinal tract in early life and develops within a year to a level close to that observed in healthy adults	[17]

Comparative assessment of faecal microbiota	*n* = 62 (i) Infants, *n* = 21, age 3 weeks to 10 months (ii) Adults, *n* = 21, age 25 to 45 years (iii) Elderly, *n* = 20, age 70 to 90 years	qPCR and 16S rRNA gene sequencing	Firmicutes to Bacteroidetes ratios (in log_10_): (i) Infants: 0.4 (ii) Adults: 10.9 (iii) Elderly: 0.6	[18]

Comparative analysis	*n* = 84 (i) Adults *n* = 20, age 25 to 40 years; (ii) Elderly *n* = 22, age 63 to 76 years; (iii) Centenarians *n* = 21, age 99 to 104 years.	16S rRNA gene sequencing	(i) Adults and Elderly: Bacteroidetes and Firmicutes dominant; low levels of Actinobacteria, and Proteobacteria (ii) Centenarians: High levels of Proteobacteria; decrease in *Clostridium *cluster XIVa, an increase in *Bacillus* species	[6]

^*∗*^Table sorted by year of publication; FISH: fluorescence in situ hybridization.

**Table 2:** Clinical studies related to variations in immunity and gut microbiota in the elderly^*∗*^.

Study details	Sample details	Methods	CFU	Outcome	Ref
Placebo controlled RCT	*n* = 24 (i) Probiotic (*L. johnsonii*) La1 (NCC533) *n = *12 (ii) Placebo (nonprobiotic) *n = *12	ELISA, C-reactive protein test, PHAGOTEST, faecal microbiota enumeration	10^9^ CFU/day/12 weeks	Daily consumption of *L. johnsonii* La1 (NCC533) may contribute to suppressing infections by improving nutritional and immunological status	[19]

RCT	*n* = 209 (i) Probiotic group: *B. longum* 2C (DSM 14579) *n* = 56, (ii) DSM 14583 *n* = 46 (iii) Placebo group *n* = 67 (iv) Control group *n* = 86	qPCR, ELISA and faecal microbiota enumeration	10^9^ CFU/day/6 months	Bifidobacterium levels in the in microbiota may be associated with change of cytokine levels	[20]

Comparative analysis	*n* = 84 (i) Adults *n* = 20, age 25 to 40 years; (ii) Elderly *n* = 22, age 63 to 76 years; (iii) Centenarians *n* = 21, age 99 to 104 years.	Tract Chip (HITChip), qPCR, 16S rRNA gene sequencing, ELISA and Flow cytometry		The proportion of centenarians showing a high inflammation score was significantly higher than in the other age groups, confirming the inflamm-ageing hypothesis	[6]

RCT	*n* = 45 (i) *B. longum* BB536 *n* = 23 (ii) Placebo (Dextrin) *n* = 22	RT-PCR, ELISA, T-RFLPs	5 × 10^10^/day/12 weeks	The potential of long-term ingestion of BB536 in increasing the cell number of bifidobacteria in intestinal microbiota and modulating immune function	[21]

Investigative RCT	*n* = 33 (*Bacillus coagulans* GBI-30, 6086 (BC30))	FISH, Gas chromatography and Flow cytometry	1 × 10^9^/day/28 days	The dietary inclusion of probiotics such as BC30 may provide a beneficial option for enhancing markers of GI health comparison with placebo	[22]

^*∗*^Table sorted by year of publication; RCT: randomized double-blinded clinical trial.
